# Impact of Repetitive Elements on the Y Chromosome Formation in Plants

**DOI:** 10.3390/genes8110302

**Published:** 2017-11-01

**Authors:** Roman Hobza, Radim Cegan, Wojciech Jesionek, Eduard Kejnovsky, Boris Vyskot, Zdenek Kubat

**Affiliations:** 1 Department of Plant Developmental Genetics, Institute of Biophysics, Academy of Sciences of the Czech Republic, Kralovopolska 135, 61200 Brno, Czech Republic; cegan@ibp.cz (R.C.); wjesionek@gmail.com (W.J.); kejnovsk@ibp.cz (E.K.); vyskot@ibp.cz (B.V.); kubat@ibp.cz (Z.K.); 2 Centre of the Region Hana for Biotechnological and Agricultural Research, Institute of Experimental Botany, 78371 Olomouc, Czech Republic

**Keywords:** sex chromosomes, transposable elements, satellites, Y chromosome

## Abstract

In contrast to animals, separate sexes and sex chromosomes in plants are very rare. Although the evolution of sex chromosomes has been the subject of numerous studies, the impact of repetitive sequences on sex chromosome architecture is not fully understood. New genomic approaches shed light on the role of satellites and transposable elements in the process of Y chromosome evolution. We discuss the impact of repetitive sequences on the structure and dynamics of sex chromosomes with specific focus on *Rumex acetosa* and *Silene latifolia*. Recent papers showed that both the expansion and shrinkage of the Y chromosome is influenced by sex-specific regulation of repetitive DNA spread. We present a view that the dynamics of Y chromosome formation is an interplay of genetic and epigenetic processes.

## 1. Introduction

The evolution of the Y chromosome architecture has historically been of interest mainly at the cytogenetic level, with a recent switch to genomics and epigenomics. The Y chromosome is a unique part of the genome since it does not recombine over some or most of its length and represents male-limited transmission [1]. The main stages of the Y chromosome evolution are manifested by an establishment of the sex-determining region, local suppression of recombination, accumulation of repeats, degeneration of genes, and shrinkage by deletions. Expansion and shrinkage are parallel processes in shaping the Y chromosome structure, varying in the impact on Y chromosome dynamics in individual stages of sex chromosome evolution [2] (Table 1). Although a number of important studies on sex determination and sex chromosome evolution have emerged recently [3,4,5], we focus mainly on the impact of repetitive DNA on Y chromosome dynamics in advanced stages of sex chromosome divergence. Since there is a lack of information in plant models possessing ZZ/ZW [6,7], we review mainly XX/XY chromosomal systems with heteromorphic and/or heterochromatin-containing sex chromosomes.

In plants, sex chromosomes have been identified in 40 species and heteromorphic sex chromosomes have been revealed in just 19 species [8,9]. Even plants with homomorphic sex chromosomes reveal structural changes in non-recombining region(s). In the case of papaya homomorphic sex chromosomes, the accumulation of repeats accounts for much of Y chromosome specific region expansion. Surprisingly, in young papaya Y chromosome (7 million years ago (mya)) at least 20 specific repeats have been identified which are absent from the autosomes and have no homology in other plant species [10]. Dynamic changes of repetitive DNA content can also influence X chromosome evolution, as shown in the case of transposable elements accumulation on the X chromosome in papaya [11]. It is not clear whether the accumulation of retrotransposing sequences on the X chromosome is a result of more efficient selection against this process in autosomes or preferential targeting to the X chromosome [12]. Interestingly, young papaya Y chromosome already reveals signatures of heterochromatization [13].

A global genomic impact of sex chromosome evolution was most studied in species with heteromorphic sex chromosomes, namely in *Cannabis sativa* (hemp) [14], *Hippophae rhamnoides* (sea buckthorn) [15], *Coccinia grandis* [16], and classical models *Rumex acetosa* (sorrel) and *Silene latifolia* (white campion) [17]. Surprisingly, even in these plants sex chromosomes are relatively evolutionarily young in comparison with many animal species—e.g., *Silene latifolia* 6 mya [18], *Rumex acetosa* 12–13 mya [19], or *Coccinia grandis* 3 mya [20].

**Figure 1:**
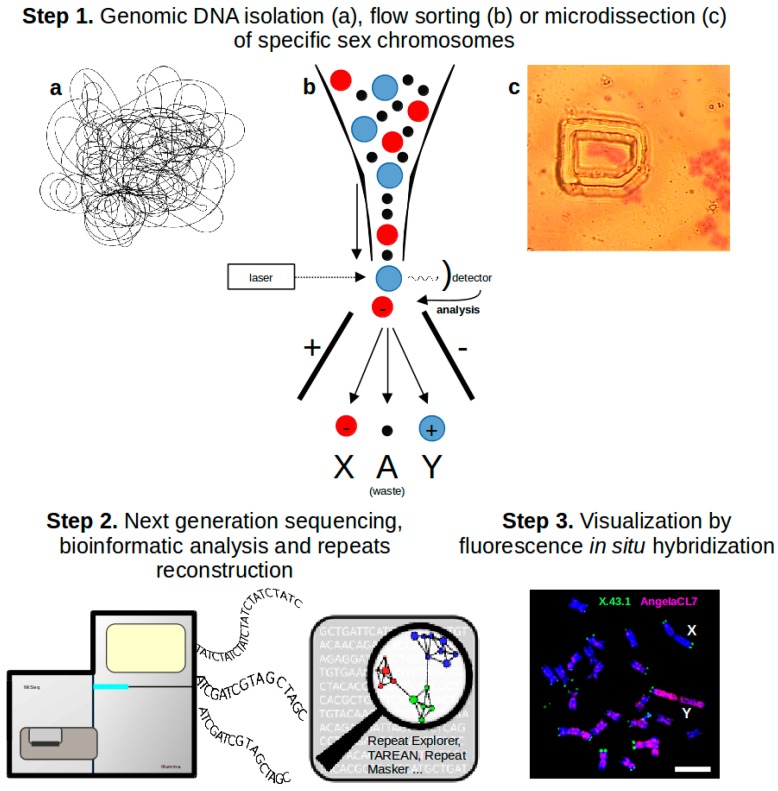
Typical workflow used for repeats reconstruction and characterization. Step 1—Genomic DNA for subsequent analysis can be isolated by three different approaches. Flow sorting and microdissection in our case are usually used for sex chromosomes separation. Step 2—Genomic DNA or separated sex chromosomes are sequenced with low coverage. Repeats are reconstructed and characterized by the clustering algorithm employed in the RepeatExplorer pipeline [22] or by the TAREAN tool [23]. Step 3—Reconstructed repeats are used as probes for Fluorescence In Situ Hybridization (FISH) localization on *Silene latifolia* metaphase chromosomes. The X and Y chromosomes are indicated, bar indicates 10 μm. Red probe illuminates the Y-biased repetitive element (Angela CL7), green probe represents the internal FISH control (subtelomeric tandem repeat X43.1).

Although none of the plant species possessing heteromorphic sex chromosomes have been sequenced yet, large-scale genome analyses have been accelerated using a combination of next generation sequencing with comparative graph-based clustering analysis of sequenced reads by RepeatExplorer pipeline [21,22]. This approach enables us to characterize the structure of the repetitive fraction of the genome without deep sequencing as well as to determine an accurate assembly of the genome (Figure 1).

In this review, we focus on the main processes that influence the heteromorphic character of sex chromosomes in plants while paying an attention to sex chromosome systems in *S. latifolia* and *R. acetosa* that represent the most studied species in this aspect.

## 2. Promiscuous DNA—Frequent Passengers Colonizing Non-Recombining Regions

Chloroplast and mitochondrial DNA sequences are frequently transferred into the nuclear genome in plants. The endosymbiotic transfer is usually dependent on recombination-based insertions of large fragments of organellar DNA into the nucleus, resulting in either nuclear insertions of plastid DNA (NUPTs) or nuclear insertions of mitochondrial DNA (NUMTs).

In plants with sex chromosomes, the chloroplast DNA has frequently accumulated on the Y chromosomes. This accumulation has been observed on the Y chromosomes of sorrel (XY_1_Y_2_ system) and white campion (XY system), being more marked in older and more degenerated Y_1_ and Y_2_ chromosomes of sorrel [24,25]. A several-fold enrichment of both chloroplast and mitochondrial sequences was also observed in the male-specific regions of the Y chromosome (MSY) and the hermaphrodite-specific region of the Y^h^ chromosome (HSY) of papaya, a plant with incipient sex chromosomes [26]. NUPTs are more frequent than NUMTs on the Y chromosome in papaya. Similar to other species possessing large Y chromosomes, organellar DNA has also accumulated in *C. grandis* [16].

## 3. Satellites and Chromatin Changes

**Figure 2:**
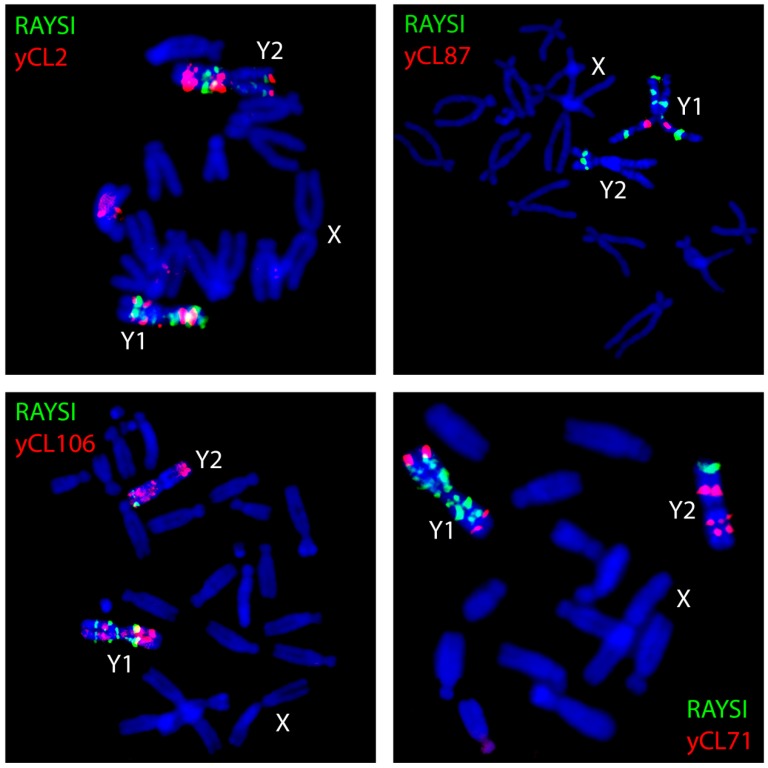
Localization of satellites on metaphase chromosomes of *Rumex acetosa* using FISH. The name of the satellite or number of satellite cluster is inside each figure.

The accumulation of satellites accompanies the evolution of sex chromosomes in some plant species with heteromorphic sex chromosomes. Satellites mostly exhibit discrete chromosomal localization. The most prominent are the heterochromatic regions in *R. acetosa* (Figure 2) that are formed by various types of tandem repeats [19,27,28,29]. In sorrel, most of the satellites show the Y-specific pattern of distribution, either exhibiting an accumulating on both Y chromosomes like RAYSI and RAYSIII [27,29], or showing the Y chromosome-specific localization such as RAYSII [27,30], indicating that the Y chromosome probably represents a suitable chromosomal context for satellite expansion. It was thought that the heterochromatic nature of Y chromosomes in *R. acetosa* reflects an advanced degeneration process, but recent results showed that a number of Y-linked genes are transcribed in this species [31]. In white campion, TRAYC and STAR-Y satellites have accumulated on the Y chromosome [32,33]. Surprisingly, these satellite regions do not correlate with heterochromatic regions in this species. This observation suggests that the accumulation of satellites predates chromatin changes in the non-recombining region of the Y chromosome.

Recently, a small Y chromosome containing several satellite DNAs and a large X chromosome resembling a mammalian sex chromosome system (rare in plants) was revealed in sea buckthorn [15]. Some satellites accumulated specifically on individual sex chromosomes (HRTR8 on the X chromosome and HRTR12 on the Y chromosome) and some satellites are shared in both X and Y chromosomes (HRTR2). Generally, the sea buckthorn genome contains an unusually larger number of satellites than the majority of plant genomes [34]. Contrary to sea buckthorn, *Coccinia grandis* has only one accumulated satellite in its entire genome, without specific pattern on the Y chromosome [16]. Taken together, satellites gather both in heterochromatic and euchromatic parts of sex chromosomes, and it remains a question as to which role they play in the heterochromatinization process.

## 4. Transposable Elements—Major Force in Sex Chromosome Dynamics

The amplification of Transposable Elements (TEs) is the dominant force increasing the plant genome size. The accumulation of retrotransposons is often responsible for the enormous size of evolutionarily young plant Y chromosomes. Various families of retrotransposons contribute unequally to the size of the X and Y chromosomes in *R. acetosa* and *S. latifolia* [18,30,35,36,37], and the proliferation of individual retrotransposons may significantly vary between closely related species with and without sex chromosomes, as was shown in *S. latifolia* and *S. vulgaris* [38] as well as in *Asparagus* [39,40]. Transposable elements thus have a strong influence on the genome architecture of evolving sex chromosomes.

**Figure 3:**
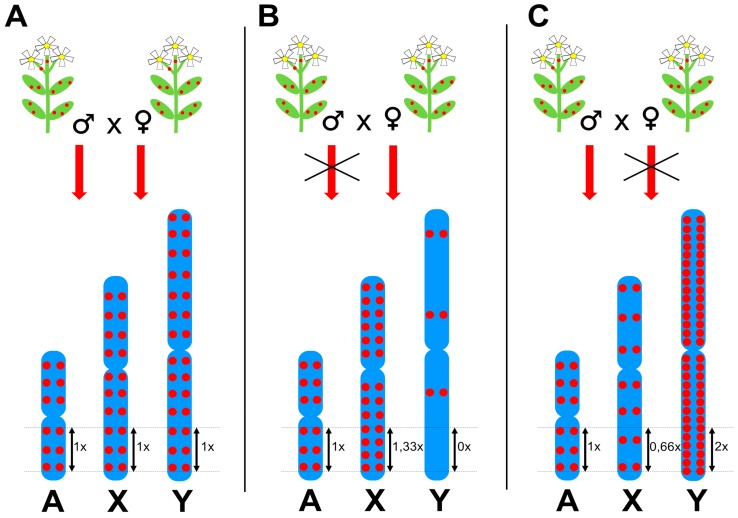
Three scenarios of transgenerational proliferation of transposable elements (TEs) in plants and their impact on the chromosomal distribution of a TE. Red dots indicate TE insertions—in blue-colored meiotic chromosomes, A-autosomes, X-chromosomes, and Y-chromosomes. Numbers next to double arrows indicate the expected density of TE insertions per one unit of length of a respective chromosome. (**A**) If a TE is passed down to offspring equally in males and females, TE insertion density is identical on all chromosomes. (**B**) If TE proliferation is disrupted in males, TE density is 1,33 times higher on the X chromosome than on autosomes, but nearly zero on the Y chromosome. (**C**) If TE proliferation is disrupted in females, TE insertion density is lower on the X chromosome compared to autosomes and twice as high on the Y chromosome than on autosomes. These three scenarios represent extreme cases of sex-specific TE activity. Real world TEs range from TEs that are almost fully sex-specifically inheritable to TEs with only slight sex-dependent inheritance.

Although some TEs prefer insertion into specific chromosomal regions such as subtelomeres, microsatellite loci [41], other transposons [42], or gene promoters [43], which probably evolved to prevent insertion into protein coding genes, most plant TEs are randomly distributed along chromosomes with different density on sex chromosomes compared to autosomes. This phenomenon is widespread and applies to the majority of highly abundant TEs in *S. latifolia* and *R. acetosa* [18,30,35,36,37]. Figure 3 shows a schematic representation of idealized chromosomal distributions of Long Terminal Repeat (LTR) retrotransposons in dioecious plants and a hypothetical causal mechanism; i.e., restricted TE proliferation in either male or female reproductive organs. Three scenarios of TE behavior can be inferred from fluorescence in situ hybridization (FISH) experiments in dioecious plants. (i) The first group of TEs are homogeneously distributed along all chromosomes, thus the average insertion density (number of insertions per unit of length) is identical for all chromosomes (Figure 3A). These TEs proliferate equally in males and females and represent a minority of TEs. (ii) The TEs of the second group are underrepresented on the Y chromosome and simultaneously their insertion density is higher on X chromosomes compared to autosomes (Figure 3B). TEs of this group vary extensively in their effect strength; some TEs are almost completely absent on the Y and strongly accumulated on X chromosomes, while others show weak underrepresentation on the Y chromosome together with a slight accumulation on X chromosomes. Such chromosomal distribution is probably caused by the prevention of TE proliferation in male reproductive cells. Although not always clearly visible in FISH data, this might be the prevalent behavior among highly abundant and transcriptionally active plant TEs, primarily Ty3/gypsy LTR retrotransposons, indicating a strong selection to inhibit most active TEs preferentially in males. (iii) TEs of the third group are accumulated on the Y chromosome and underrepresented on X chromosomes compared to autosomes (Figure 3C). Similarly to the previous case, high variability of their effect strength is observed. This behavior might be caused by TE silencing in female reproductive cells and is frequent among Ty1/copia type LTR retrotransposons, long interspersed nuclear elements (LINEs), and DNA transposons; all TEs with comparatively lower insertional activity.

Diverse TE behavior is apparently a consequence of epigenetic regulation during the development of reproductive cells and embryogenesis [18,44]. Since plants do not set aside germlines early in embryogenesis, reproductive cells differentiate from the meristematic tissues of the flower. Epigenetic marks that contributed to plant development and TE silencing have to be removed to restore totipotent state in the zygote. It is assumed that TEs make use of the opportunity of temporary deficiency in epigenetic control for transposition. However, plants have defensive mechanisms utilizing companion cells of plant gametes. While epigenetic information is gradually lost during gamete formation [45,46,47] and re-established again in the sperm and embryo, companion cells, vegetative cells in pollen and central cells in ovuli, retain low levels of heterochromatic marks, resulting in the active transcription of TEs. TE transcripts are thought to be processed to small RNA molecules that migrate to the sperm and embryo, where they reinforce both transcriptional and post-transcriptional TE silencing [48,49,50]. Epigenetic regulation is in current view a complex array of mutually interconnected pathways, sharing signal molecules such as small RNAs (sRNAs) and long non-coding (lncRNAs) as well as proteins and enzymes [51,52]. Thus, the way of particular TE silencing might be strongly individualized, which results in diverse chromosomal distribution of TEs in dioecious plants. Nevertheless, certain rules can be inferred from previous FISH experiments. The most transpositionally active TEs tend to be more intensively regulated in males, whereas less active TEs are more regulated in females. Although the clear evidence of sex-specific epigenetic regulation of TEs is still unavailable in dioecious plants, in *Arabidopsis thaliana*, a hermaphroditic model plant species, LTR retrotransposon Evade (EVD) expands if transmitted through the paternal germline but is suppressed when the active EVD element is maternally inherited [44]. It was suggested that EVD is silenced in the maternal sporophytic tissues of the flower by an epigenetic mechanism, most probably sRNA mediated post-transcriptional mRNA degradation. Such regulation of retrotransposon proliferation would result in Y chromosome expansion in the dioecious systems with XX/XY sex determination. The opposite TE behavior, female-specific transposition, has been examined in *S. latifolia*. LTR retrotransposon Ogre is the main genome size driver in *S. latifolia* [38] and is virtually absent on the Y chromosome [35,36], which is probably caused by epigenetic silencing in the sperm and embryo [18].

A crucial question resonates here: what is the reason for this apparent sex-specific epigenetic regulation of TEs? (i) Is the diverse epigenetic regulation of TEs a hit-and-miss affair? (ii) Is it an unintentional consequence of different modes of function of epigenetic mechanisms in male and female reproductive organs? (iii) Or a third explanation; is it a result of natural selection that prefers male gametes with the lowest possible number of new TE insertions, i.e. pollen grains that eliminate the most active TEs in order to decrease the risk of deleterious changes in haploid genetic information indispensable for pollen tube growths [53]? Is this a common feature of angiosperms or is it specific to evolutionarily young dioecious species possessing non-recombining Y chromosomes that still carry genes participating in pollen tube growths?

Several recent publications suggest that male reproductive organs adopted unique epigenetic pathways in *A. thaliana*. These pathways utilize micro RNAs and transfer RNA (tRNAs) for the efficient silencing of TEs in pollen grains [50,54]. Namely, tRNA-derived sRNAs were proved to target mainly Ty3/gypsy LTR retrotransposons, dominant TEs in dioecious plants. Thus, the male germline might possess a reinforced epigenetic barrier against TEs, a barrier that might point to the most active TEs often underrepresented on Y chromosomes in dioecious species. It is difficult to monitor long-term effects in hermaphroditic species without sex-specific DNA regions (sex chromosomes), but dioecious plants provide a plethora of TEs showing signs of sex-specific regulation. Nevertheless, without deep studies of long-term TE behavior in hermaphrodites, we cannot conclude whether those enigmatic distributions of TEs on sex chromosomes are a consequence of common mechanisms in reproductive organs or a consequence of the independence of individual sexes at the genome level that lead to the evolution of specific regulatory mechanisms shaping the architecture of male and female genomes.

## 5. Unusual DNA Structures on the Y Chromosome and Their Possible Function

Human and chimp Y chromosomes contain large palindromes in their non-recombining region [57,58] containing genes important for sex determination. Palindromes enable intrachromosomal gene conversion and thus help to eliminate deleterious mutations and protect the Y chromosome against degeneration [57,59,60]. Although the large palindromes present on the Y chromosome have different behavior compared to their shorter counterparts, these motifs generally readily form DNA hairpins that belong to non-B DNA conformations together with e.g., triplex and quadruplex DNA. An important role of these structures in the function and evolution of genomes is now only being recognized. For example, it has been suggested that palindromes on the Y chromosome (and on the X chromosome) favor the formation of unusual chromatin structures such as cruciforms [59], and that this conformation regulates the expression of palindrome genes by avoiding transcriptional inactivation [61,62]. Unfortunately, the complete sequence of a plant Y chromosome is not available, yet and the information of whether the palindromes are present also in plant Y chromosomes is still missing.

Recent studies showed that unusual DNA conformations are present not only in genes and their regulatory regions, but also within transposable elements [63,64]. The sequence motifs forming quadruplexes are often located within LTRs of plant LTR retrotransposons, and the higher abundance of quadruplexes within evolutionarily younger retrotransposon suggests their functional role [63,64]. Since these quadruplexes can represent the hot-spots of recombination, it would be interesting to know whether their abundance on the non-recombining Y chromosome is different compared to the rest of the genome.

The activity of LTR retrotransposons on the Y chromosome is regulated by a number of factors—not only by regulatory motifs within the elements (e.g., PBS – primer binding site, PPT – polypurine tract, psi element), but also by epigenetic mechanisms [56,65]—e.g., the Y chromosome of *R. acetosa* is formed by heterochromatin [66]. Moreover, DNA conformation can also affect TE activity [67], and the secondary DNA structure thus represents another factor playing a role in the life cycle of transposable elements and involves not only transcription and translation but also the reverse transcription and integration phases.

The Y chromosomes of several plant species (*S. latifolia*, *R. acetosa*) accumulate microsatellites that are known to readily adopt unusual DNA conformation like e.g., A-DNA or Z-DNA [68,69]. Such DNA with changed conformation is often nucleosome-free [70] and represents a preferred target for transposable elements insertions. Therefore, microsatellites are often more common in the neighborhood of transposable elements [69,71]. Moreover, DNA conformation plays a role also in microsatellite expansion when hairpins are formed during replication slippage, resulting in an increase in the number of monomer units [72].

The unique feature of the Y chromosomes is the reduced rate of recombination, but some recombination processes are still in action there. In this low- or no-recombination context, the strong recombination activity evolved in the form of gene conversion [58], homogenizing the large palindromes on the human and chimp Y chromosomes, and a similar process is expected to be present in the evolutionarily younger plant Y chromosomes. Then, the Y chromosome represents a mosaic of regions shaped by two opposing evolutionary dynamics: (i) the decay of genes and suppressed recombination in X-degenerate regions and (ii) gene acquisitions and the activation of intra-chromosomal recombination in palindromic (ampliconic) regions [59]. Transposable elements and satellites that form a majority of the Y chromosomes are also suitable targets for gene conversion [73,74].

Moreover, TEs and satellites are, thanks to their repetitive nature, good subjects for another recombination process—ectopic recombination. The turnover of transposable elements in plant genomes is much higher than in animals [55]. Ectopic recombination between long terminal repeats of the same retrotransposon results in the deletion of an internal part of LTR retrotransposons, leaving behind “solo LTRs”. Similarly, a region located between two elements can be deleted by ectopic recombination, leading to genome size reduction [75]. Since the recombination is lower on the Y chromosome than on other chromosomes, and the downsizing is less efficient, one can expect a lower proportion of solo LTRs compared to full-length elements on the Y chromosome.

## 6. Conclusions

Although the advent of genomic methods has shed light on many aspects of heteromorphic sex chromosome formation in dioecious plants, there is still limited information about the impact of structural changes on the function of sex-linked genes. Transposable elements can affect sex chromosome evolution directly via insertion into a specific site, and indirectly by affecting the expression of closely linked genes by epigenetic mechanisms. Large-scale genomic response to repetitive DNA accumulation results in changes in chromatin status, which can in some species lead to heterochromatinization. Is an elevated rate of transposon accumulation the cause or consequence of sex chromosome degeneration? How much does cross-talk of transposable elements with genic regions affect dosage compensation evolution? How much are epigenetic processes involved in the degeneration of sex chromosomes? Surprisingly, structurally divergent sex chromosomes in *S. latifolia* are euchromatic while papaya homomorphic sex chromosomes reveal clear signs of heterochromatization. It is likely that sex chromosome evolution is affected by a number of mechanisms that vary in individual dioecious species such as population size, genome dynamics, regulation of TEs, etc. It remains to be answered which processes are shared among the species and which mechanisms are unique in individual species. Recent studies clearly show that plants possessing sex chromosomes can regulate the activity of TEs and subsequently regulate their spread in non-recombining regions. Whether this phenomenon is specific for dioecious plants or it is a common attribute of angiosperms remains to be elucidated. It is tempting to speculate that not only RNAi (RNA interference) machinery but also specific DNA conformation such as quadruplexes may play a role in the dynamics of the spread of repetitive elements within sex chromosomes.

## Figures and Tables

**Table 1:** Summary of processes forming sex chromosome structure in the expansion and shrinkage phases.

Mechanism	References
***Expansion phase***	
Satellites expansion	Sousa et al. (2016) [16], Shibata et al. (1999) [26],
	Kubat et al. (2008) [18], Puterova et al. (2017) [15],
Retrotranspositions	Na et al. (2014) [10], Cermak et al. (2008) [34],
	Sousa et al. (2016) [16]
Promiscuous DNA insertions	VanBuren et al. (2013) [25], Kejnovsky et al. (2006) [23]
***Shrinkage phase***	
Ectopic recombination	Kejnovsky et al. (2009) [12]
Deletions	Hawkins et al. (2009) [55]
***Both expansion and shrinkage phase***	
Epigenetic regulation of TEs	Kubat et al. (2014) [18]
Chromatin modification	Zhang et al. (2008) [13], Lengerova et al. (2001) [56]

TE: Transposable element.
